# Environmental Toxins Found Historically in the Polycythemia Vera Cluster Area and their Potential for Inducing DNA Damage

**DOI:** 10.4172/2161-0525.1000551

**Published:** 2021-02-15

**Authors:** EA Irvin-Barnwell, KM Benson, M Lu, A Ragin, J Wheeler, R Hoffman

**Affiliations:** 1 Agency for Toxic Substances and Disease Registry, Atlanta, GA, USA; 2 Icahn School of Medicine, Mt. Sinai, New York, NY, USA

**Keywords:** Myeloproliferative neoplasms, Polycythemia vera, Comet assay, *In vitro* assays

## Abstract

In 2006, the Agency for Toxic Substances and Disease Registry received a request to determine whether a cluster of polycythemia vera patients existed in a northeast Pennsylvania community. A significant cluster of PV cases was identified at the nexus of three counties near several hazardous waste sites. The current study evaluated the potential for a select number of environmental contaminants previously detected in the cluster area to induce DNA damage using *in vitro* assays with hematopoietic stem-cell derived progenitor cells.

CD34+ cells were isolated from normal cord blood samples and were cultured for 48–72 hours to generate erythroid progenitor cells. Eighteen compounds were chosen for the assay; arsenic trioxide, benzo(a)pyrene, benzene, methylene chloride, 2,3,7,8-tetrachlorodibenzo-*p*-dioxin (TCDD), trichloroethylene, potassium chloride, ethylbenzene, benzo[k]fluoranthene, styrene, cadmium chloride, hydroquinone, 1,1,1-trichloroethane, sodium cyanide, manganese chloride, chromium oxide, lead oxide, and sodium arsenite. Genotoxicity of the compounds was determined using the comet assay, and toxicity determined via the cell viability assay.

Using the comet assay, 16 compounds at 10 nM concentration, induced a significant amount of DNA damage compared to the control. When evaluating whether a dose-dependent relationship was present, seventeen of the eighteen compounds led to greater DNA damage with increasing exposure concentrations. 2,3,7,8-TCDD was particularly potent, inducing DNA damage in virtually all cells at 1 μM. In conclusion, most of the toxins evaluated using the comet assay showed potential to induce DNA damage in hematopoietic cells, and the genotoxic effects were dose-dependent.

## Introduction

In 2005, the Pennsylvania Department of Health requested the assistance of the Agency for Toxic Substances and Disease Registry (ATSDR) in studying the pattern of polycythemia vera (PV) in an area in northeast Pennsylvania, referred to as the tri-county area. The request came after local physicians and community members raised concerns regarding the diagnosis of 4 cases of PV on a rural road in the tri-county area. Concerns also included possible historical and current exposure from a nearby *National Priorities List* (NPL) site, one of seven in the tri-county area.

PV is a myeloproliferative neoplasm (MPN) of the bone marrow characterized by erythrocytosis [1,2]. MPNs also include essential thrombocytosis (ET) and primary myelofibrosis (PMF). In 2005, researchers identified a somatic mutation, JAK2V617F, found in approximately 97% of PV cases [3]. The etiology of both the mutation and disease remains unknown. Using the JAK2V617F mutation test, ATSDR confirmed 33 PV cases in the tri-county area and identified a statistically significant cluster at the nexus of the three counties [2]. As a result of the initial investigation, ATSDR established a multi partner research portfolio to continue the PV investigation. The purpose of the current study was to evaluate the potential for contaminants known to be present historically within the tri-county area to cause DNA damage using *in vitro* assays.

## Methods

Low density mononuclear cells (MNC) were isolated from normal cord blood collections and erythroid progenitor cells were cultured from isolated CD34+ cells. Eighteen compounds were chosen based on their historical presence in the tri-county area: arsenic trioxide, benzene, benzo(a)pyrene, benzo[k]fluoranthene, cadmium chloride, chromium oxide, ethylbenzene, lead oxide, hydroquinone, manganese chloride, methylene chloride, potassium chloride, sodium arsenite, sodium cyanide, styrene, 2,3,7,8-tetrachlorodibenzo-p-dioxin (TCDD), trichloroethylene (TCE), and 1,1,1-trichloroethane. The compounds were evaluated using a cell viability assay and Comet assay at varying concentrations (10 nM, 100 nM, and 1000 nM). Statistical analyses were performed using SAS 9.3 (SAS Institute Inc., Cary, NC). Proc GLM and SNK tests were used to determine increases in DNA damage; statistical significance was set at 0.025 to avoid Type 1 error.

### Comet assay

The Cell Biolabs OxiSelect^™^ Comet Assay kit (Cell bioLabs, Inc) was used. After treatment with each individual compound for 24 hours, cells were mixed with molten agarose before application to the OxiSelect^™^ Comet Slide. Embedded cells were treated with a lysis buffer and alkaline solution. Samples were electrophoresed in a horizontal chamber to separate intact DNA from damaged fragments and the assay repeated in triplicate for each dose. Following electrophoresis, samples were dried, stained with a DNA dye, and visualized. Fluorescent images were acquired using a IX71 fluorescence microscope and MicroSuite software.

### Cell viability assay

In order to verify the effects of toxic compounds on cell viability, erythroid progenitor cells were incubated with each compound at 10, 100, 1000 nM. Briefly, 1 × 10^5^ cells/well were seeded into each well of a 24-well plate, and cultured with IMDM containing 10% of FBS, 50% of SCF and 2 U of EPO, to which each individual compound was added and incubated for 4 days. Cell viability was determined using a 0.4% trypan blue solution. The assay was performed in triplicate for each chemical at each dose.

## Results and Discussion

All chemicals were evaluated at the three different concentrations, 10 nM, 100 nM, 1000 nM using the Comet assay. Previous research has shown the comet assay is a well-established sensitive method for detecting genotoxicity in mammalian cells [4–6]. Statistical significance was set at p-value=0.01 to adjust for multiple comparisons. At the 10 nM concentration, arsenic trioxide, benzo(a)pyrene, potassium chloride, benzo(k)fluoranthene, styrene, cadmium chloride, 1,1,1-trichloroethane, TCDD, methylene chloride, sodium cyanide, benzene, chromium oxide, lead oxide, and sodium arsenite generated significantly greater numbers of cells with comets than the control. At 100 nM, benzo(a)pyrene, potassium chloride, benzo(k)fluoranthene, styrene, hydroquinone, 1,1,1-trichloroethane, TCDD, TCE, sodium cyanide, manganese chloride, and chromium oxide were significantly different from the control. At the highest concentration (1,000 nM), arsenic trioxide, benzo(a)pyrene, potassium chloride, styrene, cadmium chloride, hydroquinone, 1,1,1-trichloroethane, TCDD, TCE, methylene chloride, sodium cyanide, benzene, chromium oxide, lead oxide, and sodium arsenite were significantly different from the control. Chromium oxide, sodium cyanide, TCDD, 1,1,1-trichloroethane, styrene, potassium chloride, and benzo(a)pyrene were significantly different from the control at all concentrations. A dose-dependent relationship for benzo(a)pyrene, potassium chloride, styrene, cadmium chloride, hydroquinone, TCDD, TCE, methylene chloride, benzene, lead oxide, and sodium arsenite and DNA damage was found. Similarly, studies using the comet assay have shown many of these contaminants can induce DNA damage including arsenic trioxide, benzene, lead, cadmium, ethylbenzene, TCE, and styrene [7–11]. However, the literature is sparse on any associations between chemicals exposures and PV. An in-depth review by Anderson et al. cited two studies that show an increased risk of MPNs including PV in persons exposed to benzene or petroleum [12–14].

Concerning the cell viability assay, seventeen compounds resulted in significantly decreased cell viability in a dose-dependent manner (Table 1). For benzene, cadmium chloride, chromium oxide, potassium chloride, lead oxide, and sodium cyanide, cell viability was significantly decreased at increasing concentrations. Using the Comet assay and the cell viability assay, treatment with 1,000 nM TCDD resulted in 100% cell death and 100% of cells having DNA damage.

Using the Comet assay, the majority of the contaminants induced DNA damage. Similarly, cell viability decreased after treatment with a contaminant at concentrations of 100 nM and 1000 nm for all except TCE. For both the Comet assay and cell viability assay, the genotoxic effects of the compounds were dose-dependent. Interestingly, 2,3,7,8-TCDD was particularly toxic, inducing DNA damage and cell death in virtually all cells at a concentration of 1,000 nM.

To determine comparability of chemical concentrations in the current study and historical environmental contamination, we used information from the ATSDR PV environmental database to determine maximum historical groundwater concentrations in the tri-county area. Most of the chemicals had historical groundwater concentrations lower than concentrations used in the assays. For example, 1000 nM of styrene is approximately 104.14 μg/L which is 9.5 times greater than the historically present 11 μg/L. Similarly, 1000 nM of benzo(a)pyrene is approximately 252.31 μg/l, much larger than the historically present 4 μg/L in groundwater. However a few chemicals had higher concentrations in the tri-county area, including benzene with a maximum concentration of 3430 μg/L compared to the 1000 nM (78.1 g/mol) concentration used in the assay.

The present study does have limitations. Specifically, the concentrations used in the current study might not be indicative of actual historical exposure levels to residents in the tri-county area. Additionally, extrapolating doses from *in vitro* testing to environmental exposure is fraught with uncertainties. While the present study indicates that some of the chemicals historically present do have genotoxic effects, we could not determine whether the genotoxic effects played a role in the creation of the cluster of PV patients. Future research could involve calculating target levels (mononuclear intracellular levels) using recent advances in PBPK modeling to help elucidate these findings.

## Figures and Tables

**Table 1:** Percent of non-viable cells with standard deviation after treatment with the environmental contaminants as shown by the cell viability assay.

Percent of non-viable cells (%)			
Chemical	10 nM (SD)	100 nM (SD)	1000 nM (SD)
Arsenic trioxide*	5.00 (7.00)	32.33 (11.37)	54.33 (6.03)
Benzo(a)pyrene*	1.00 (1.73)	42.67 (13.28)	64.00 (8.72)
Potassium chloride*	0.00 (0.00)	42.00 (12.12)	79.67 (1.53)
Ethylbenzene*	0.00 (0.00)	38.00 (7.21)	10.00 (10.00)
Benzo[k]fluoranthene*	2.67 (4.61)	38.00 (16.37)	59.00 (8.66)
Styrene*	14.33 (15.31)	51.00 (9.17)	55.33 (3.21)
Cadmium chloride*	2.67 (4.62)	42.33 (7.37)	86.33 (0.58)
Hydroquinone*	1.00 (1.00)	50.67 (5.77)	51.00 (9.00)
1,1,1-Trichloroethane*	7.00 (5.57)	46.00 (6.24)	61.00 (3.46)
TCDD*	13.33 (14.47)	44.33 (11.93)	100.00 (0.00)
TCE	9.00 (7.94)	27.33 (11.93)	38.33 (4.51)
Methylene chloride*	0.00 (0.00)	32.33 (19.76)	45.00 (1.00)
Sodium cyanide*	0.00 (0.00)	36.33 (8.02)	55.00 (2.00)
Manganese chloride	3.33 (4.16)	41.00 (3.61)	36.33 (2.08)
Benzene*	9.00 (7.81)	29.33 (2.31)	48.33 (1.15)
Chromium oxide*	1.33 (2.31)	25.00 (5.00)	44.00 (2.65)
Lead oxide*	3.33 (4.16)	31.67 (1.15)	51.00 (9.00)
Sodium arsenite*	20.67 (15.31)	23.67 (2.08)	65.00 (5.57)

* The percent of non-viable cells significantly increased as the dose increased.

## Abbreviations:

ATSDR: Agency for Toxic Substances and Disease Registry
MPN: Myeloproliferative Neoplasm
PV: Polycythemia Vera
ET: Essential Thrombocytosis
PMF: Primary Myelofibrosis
TCDD: 3,7,8-tetrachlorodibenzo-p-dioxin
TCE: Trichloroethylene
NPL: National Priorities List
MNC: Mononuclear Cells
